# Findings supporting neonatal screening for sickle cell disease: an observational study in Senegal

**DOI:** 10.3389/fped.2025.1578570

**Published:** 2025-05-29

**Authors:** Lucie Petigas, Ndiogou Seck, Dominique Doupa, Ibrahima Diagne, Matthias Roth-Kleiner

**Affiliations:** ^1^ Faculty of Biology and Medicine, University of Lausanne, Lausanne, Switzerland; ^2^ Center for Research and Ambulatory Management of Sickle Cell Disease (CERPAD), St. Louis, Senegal; ^3^ Department of Pediatrics, Faculty of Health Sciences, Gaston Berger University, Saint-Louis, Senegal; ^4^ Directory of the CERPAD Laboratory, St. Louis, Senegal; ^5^ Medical Directory, University Hospital of Lausanne (CHUV), Lausanne, Switzerland

**Keywords:** neonatal screening, sickle cell disease, hemolytic anemia, Senegal, health disparities

## Abstract

**Introduction:**

Sickle cell disease (SCD) is a major contributor to morbidity and mortality in sub-Saharan Africa, and early detection through neonatal screening can improve outcomes. In Senegal, systematic screening is not yet implemented. This study describes two cohorts of children diagnosed with SCD: those identified through neonatal screening and those diagnosed clinically after presenting symptoms.

**Methods:**

This retrospective study involved two cohorts of children diagnosed with SCD in St. Louis, Senegal, between 2010 and 2020—one through neonatal screening (A) and the other clinically (B). Epidemiological, clinical, and management data were analyzed.

**Results:**

Cohort A included 17,083 screened infants (74% screening rate), with 40 diagnosed at a mean age of 70.48 days, showing low complication rates and requiring less intensive treatment. Cohort B, with 39 clinically diagnosed children, had a mean diagnosis age of 21.9 months, with higher rates of hospitalizations, transfusions, and acute anemia. Vaccination and antibiotic prophylaxis were high in both cohorts.

**Discussion:**

Neonatal screening enables early diagnosis, reducing complications and enabling timely interventions, while children diagnosed after symptoms face more severe disease. Early genetic counseling and addressing consanguinity are key for better outcomes. Challenges such as limited funding, equipment, and trained personnel must be addressed for broader implementation.

**Conclusion:**

Neonatal screening aligns with public health goals by reducing morbidity and mortality, and the long-term economic burden on families and healthcare systems. It is particularly relevant in the context of increasing global migration patterns, underscoring the need for such programs worldwide.

## 1 Introduction

Sickle cell disease (SCD) is a hemoglobinopathy caused by the presence of hemoglobin S, inherited in an autosomal recessive pattern. It includes various genotypes (SS, SC, SD, Sβ and others), each with distinct clinical outcomes. While individuals with AS heterozygosity generally exhibit no clinical manifestations, those with SC disease demonstrate an intermediate clinical severity when compared to the more severe complications associated with SS homozygosity (1). In heterozygous Sβ-thalassemia, the severity of the condition is influenced by the specific severity of the beta-thalassemia mutation (β^+^ or β⁰) (2). Under low oxygen conditions, hemoglobin S polymerizes, deforming red blood cells, which leads to vaso-occlusive crises (VOC) and hemolytic anemia. Symptoms typically don't appear before three months of age due to the presence of a high percentage of fetal hemoglobin (HbF) (3, 4).

Clinically, patients present with varying degrees of anemia, sometimes with jaundice and growth delays. VOCs are triggered by infection, dehydration, cold temperatures, intense exercise, and high altitude (3, 4). SCD is a chronic condition for which curative therapies exist (e.g., stem cell transplantation, gene therapy), but their use varies between settings of care. Management includes preventive measures like vaccinations, antibiotic prophylaxis, and hydroxyurea, alongside symptomatic care. In resource-limited settings, interventions focus on pain management and blood transfusions. The burden on families is significant, especially when multiple children are affected (5).

Epidemiologically, the WHO estimates that over 300,000 children are born annually with severe SCD, mostly in sub-Saharan Africa (6, 7). The sickle cell trait (HbAS) provides protection against malaria, influencing its distribution (8). Senegal has a 10%–11% prevalence of the sickle cell gene (4). In contrast, Europe's prevalence is around 0.03% (9). According to UNICEF, the under-five mortality rate in central and western sub-Saharan Africa was approximately 88.57 per 1,000 live births in 2022 (10). Acute anemia and infections are leading causes of this high mortality rate among infants and young children beyond the neonatal period (7). The World Health Organization (WHO) estimates that SCD accounts for up to 15% of these deaths in children under five across Africa. Without early diagnosis and treatment, 50%–80% of children with SCD in Africa may die before the age of 5 (5). In Senegal, there is limited clinical data on young children with SCD, partly due to the high early mortality rate and the frequent late diagnosis of the condition. Indeed, the average age of diagnosis is 4 years and 11 months and of their first follow-up consultation is 6 years and 2 months (10).

SCD meets the WHO's screening criteria, adapted from the Wilson and Jungner framework (11). These criteria imply that the disease must represent a significant public health problem; there must be interventions with a real impact on the disease, with available and acceptable treatments; the patients to be treated must be able to be identified and an appropriate diagnostic test must be available and accepted by the population. Furthermore, the natural history of the disease should be known. There must be an early latent phase, and the costs and inconveniences associated with screening should be outweighed by the benefits. These criteria support the effectiveness of neonatal screening and related prevention programs for managing the disease (9, 10).

Methods for screening include capillary electrophoresis, isoelectric focusing (IEF), and high-performance liquid chromatography (HPLC), but these techniques require expensive equipment (12). Mass spectrometry (MS) has emerged as a powerful alternative, offering rapid, sensitive, and highly quantitative detection of hemoglobin variants, including hemoglobin S, in dried blood spot samples. This approach is particularly advantageous for newborn screening, as it facilitates safe and accessible diagnostics both locally and globally (13). Point-of-care tests (POCT) like HemoTypeSC (Silver Lake Research Corporation, Azusa, USA), Gazelle (Hemex Health, Portland, USA), and Sickle SCAN® (BioMedomics, Durham, USA) offer cost-effective alternatives, suitable for resource-limited areas (14). These different tests differ in their analytical capacity to detect specific hemoglobin (Hb) variants. Both HemoTypeSC and Sickle SCAN® are lateral flow immunoassays based on antigen-antibody interactions, enabling the qualitative detection of HbA, HbS, and HbC, and thereby allowing for the identification of common phenotypes such as HbAA, HbAS, HbSS, HbAC, HbSC, and HbCC. However, they don't detect HbF, which significantly reduces their diagnostic reliability in neonates, in whom HbF predominates and may mask the presence of pathological variants. In contrast, the Gazelle platform, which employs microchip-based electrophoresis, enables the detection of HbA, HbS, HbC, and HbF. This broader detection profile makes Gazelle more suitable for newborn screening, as it can differentiate between normal and pathological hemoglobin patterns even in the context of elevated HbF. Furthermore, Gazelle provides quantitative results, which can aid in the differentiation of complex genotypes (e.g., HbSS vs. HbS/β⁰-thalassemia) and can be used to monitor therapeutic response, particularly in patients receiving hydroxyurea therapy, where HbF levels serve as an important biomarker (15).

Neonatal screening for sickle cell disease has been implemented in countries with high human development index while its implementation remains limited in high-prevalence, low-resource settings. In the United States, universal newborn screening for SCD has been mandated in all states since the early 2000s, leading to significant reductions in childhood morbidity and mortality through early diagnosis and timely interventions such as penicillin prophylaxis and vaccination programs (16). Similarly, Spain has introduced systematic screening in regions with high-risk populations, such as Catalonia, where a program implemented between 2015 and 2022 demonstrated effective early detection and linkage to care (17). Neonatal screening programs vary across Africa. The Consortium on Newborn Screening in Africa (CONSA), led by the American Society of Hematology, has established a collaborative platform across several African countries to implement and evaluate newborn screening and early intervention strategies, marking a significant step toward sustainable and continent-wide SCD care (18). Nigeria, with 150,000 children born annually with SCD, has implemented neonatal screening programs, although these are not uniform nationwide, yet (19). Ghana, with a 2% prevalence of children with SCD, has been developing a neonatal screening program since 1995 (20). In Ivory Coast and Mali, where the prevalence is around 12%, pilot neonatal screening programs are also in development (19).

Despite its demonstrated relevance, SCD is not systematically screened for in Senegal. Barriers such as the lack of reliable and recent data on the morbidity and mortality of children with SCD in Africa, funding, availability for equipment, and trained personnel hinder systematic screening (5, 21). In this context, the Center for Research and Ambulatory Management of Sickle Cell Disease (CERPAD: Centre de recherche et de prise en charge ambulatoire de la drépanocytose) was established in 2015 to pilot a neonatal screening model. Its goal is to assess the benefits of early SCD care, improve children's outcomes, and create a systematic screening model (22, 23).

This study aims to describe the follow-up of two cohorts of children, one managed following neonatal screening (A) and a second with diagnosis during a symptomatic episode (B), to assess the clinical evolution of children that did not benefit from early follow-up, to explore factors in favor of neonatal screening, and to assess the feasibility of systematic screening in Senegal. We also aimed to gain epidemiological information through the analysis of data collected during systematic screening conducted in the two largest maternity wards of St-Louis.

## 2 Methods

This retrospective observational study involved two cohorts of children diagnosed with SCD.

Cohort A includes children diagnosed via neonatal screening, born between July 1, 2017, and December 31, 2020, at the two main maternity facilities in St. Louis, Senegal, the maternity ward of the Regional Hospital Center of St. Louis (CHR-SL) and the Saint-Louis Reference Health Center. Screening occurred before discharge from the maternity ward, during hospitalization in neonatology, or at postnatal visits on the 8th or 15th day for those not previously screened. With parental consent, heel-prick blood samples were collected on Guthrie-type filter paper. These samples were analyzed using IEF, with additional analyses by HPLC or capillary electrophoresis to confirm the diagnosis. Data from these children were compiled from computerized and paper records, covering July 2017 to April 2021. Four siblings identified through family screening were excluded.

Cohort B consists of children diagnosed through clinical signs or family investigations, born between May 1, 2010, and April 30, 2019. Data collection spanned from March 2012 to November 2020, focusing on information from birth until the age of 5, including follow-up information up to April 2021. Data for cohort B were collected from the paper records at the CHR/SL.

Children of cohort A were followed at CERPAD, with regular monitoring intervals based on age. Standard follow-up intervals were monthly for children under 1 year, bi-monthly for those aged 1–4 years, and quarterly from 4 years onward. Children of cohort B were followed at Regional Hospital Center of St-Louis (CHR/SL) and offered trimestriel checkups (clinical and biological). Children of both cohorts were vaccinated according to the same schedule as other children until 15 months, following Senegal's Expanded Programme on Immunization (EPI) (24). Thereafter, Hexaxim was proposed at 18 months, followed by Typhim VI, Menactra, and Pneumo 23 from the age of 2, with boosters every 3–5 years. The costs of the additional vaccinations for cohort A were covered by the CERPAD project and were assumed by the families for children of cohort B. Antibiotic prophylaxis with amoxicillin was recommended for children under 5 years of age as soon as the diagnosis was established, given that penicillin is frequently unavailable in Senegal. This prophylaxis remained indicated until the age of 5. The associated costs were covered following the same coverage modalities as those described for vaccination.

Parental consent for cohort A was obtained, and ethical approval for both cohorts was granted by the National Health Research Ethics Committee (CNERS), through the research protocol SEN2022/144.

The study used epidemiological, clinical, biological, and management-related data, coded into Excel and analyzed using SPSS [version 29.0.1.0 (171)]. We analyzed clinical findings when they were documented in the child's file. However, we lacked detailed information about the specific age at which these findings occurred (under 5 years old). Variables were compared using chi-squared tests when all expected variables were ≥5. Fisher's exact test was used in cases of small sample sizes or when one or more expected counts were <5, particularly for rare phenotypes or outcomes with low frequencies (e.g., SC, Sβ, mother's phenotype). No statistical comparisons were performed when there was no variability between groups (e.g., 100% or 0% in both cohorts, such as father's phenotype). Missing data were documented where relevant. Statistical significance was defined as *p* < 0.05.

## 3 Results

### 3.1 Cohort A

**Figure 1:**
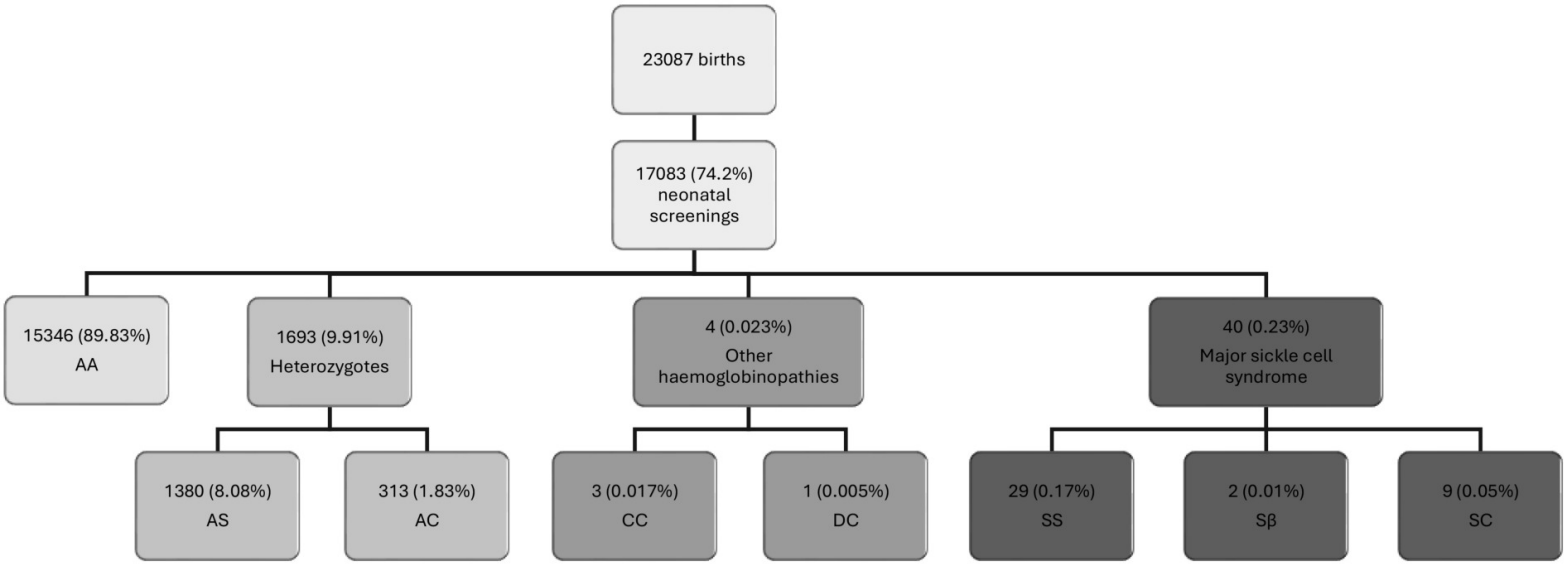
Results of sickle cell disease screening in a neonatal population conducted at CERPAD^a^, St. Louis, Senegal. ^a^Data collected by the Center for Research and Ambulatory Management of Sickle Cell Disease (CERPAD) of St-Louis, Senegal, between April 1, 2017 and April 30, 2021, at the two main maternity facilities in St. Louis, Senegal, the maternity ward of the Regional Hospital Center of St. Louis (CHR-SL) and the Saint-Louis Reference Health Center.

Cohort A consists of children diagnosed during the neonatal screening between July 1, 2017, and December 31, 2020. Of the 23,087 live births, 17,083 infants were screened, representing a screening rate of 74%. Among the screened newborn population, we found 40 children with genetic traits of severe SCD. Phenotypic distribution was as follows: 72.5% (29) SS, 22.5% (9) SC, 5% (2) Sβ (Figure 1). Other hemoglobin anomalies were identified in 3 cases (0.02%) with the CC phenotype, and in 1 case (0.005%) with the DC phenotype. 1,380 (8.1%) children had a heterozygote AS phenotype and 313 cases (1.83%) a heterozygote AC phenotype representing asymptomatic carriers.

Although the clinical manifestations of the different beta thalassemia genotypes (β^+^ and β⁰) can vary significantly, the specific genotype could not be identified due to insufficient detail in the available data. Additionally, 4 children were diagnosed through family screening as part of the CERPAD follow-up for children diagnosed at birth. The data for these 4 children were excluded from the results since they were not diagnosed at birth without the possibility of early follow-up.

All children had at least one consultation. Sometimes, there were no subsequent consultations to characterize their evolution.

Parental knowledge of their own sickle cell status was reported by 12.5% upon the birth of their child with SCD. Data were collected from 37.5% of fathers and all had an AS profile. All mothers but one could be tested and they all showed an AS profile. Consanguinity was noted in 50% of parental couples to varying degrees. Parents had an average of 2.83 children, and 35% of the children also had at least one sibling with SCD (Table 1).

**Table 1:** Descriptive data of children diagnosed with sickle cell disease at birth (A) and after a symptomatic episode (B).

Cohort		A^a^	Missing data	B^b^	Missing data	*p*-value^f^
*N* (%)		40 (100%)	0	39 (100%)	0	NA
Sexe—F		27 (67.5%)	0	15 (38.5%)	0	**0.0183**
Mean inclusion age (days)		70.48 (2.32 months)	0	666.6 (21.9 months)	0	**<0.0001**
Phenotype	SS^c^	29 (72.5%)	0	37 (94.9%)	0	**0.0129**
	SC^c^	9 (22.5%)	0	1 (2.6%)	0	**0.0143**
	S*β*^c^	2 (5%)	0	1 (2.6%)	0	NA
Father's status	Heterozygotic AS	15 (100%)	25	12 (100%)	27	NA
Mother's status	Heterozygotic AS	40 (100%)	1	18 (46.2%)	19	0.1073
	Homozygotic SS	0 (0%)	1	2 (5.1%)	19	NA
Parental knowledge of their status^d^		5 (12.5%)	0	12 (30.8%)	0	0.0888
Consanguinity between parents^e^		20 (50%)	0	24 (61.5%)	0	0.4204
>1 child with sickle cell disease in family		14 (35%)	0	14 (36.8%)	1	NA

^a^ Cohort A: children diagnosed at birth by general screening and followed in the Center for Research and Ambulatory Management of Sickle Cell Disease in St-Louis, Senegal.

^b^ Cohort B: children diagnosed after a symptomatic episode consulting to Regional Hospital Center of St-Louis, Senegal.

^c^ SS = homozygous genotype composed of two haemoglobin S alleles, SC = heterozygous genotype composed of one haemoglobin S allele and one haemoglobin C allele, Sβ = heterozygous genotype composed of one haemoglobin S allele and one haemoglobin β allele.

^d^ Parental knowledge of their own status before birth of their child diagnosed with sickle cell disease.

^e^ Parental consanguinity up to the third degree observed in a child with sickle cell anemia.

^f^ *p* < 0.05 indicating statistical significance, are shown in bold.

As additional comorbidities, 6 children had malnutrition, 7 had known atopy or asthma, 2 had cardiac pathologies,1 had epilepsy.

**Figure 2:**
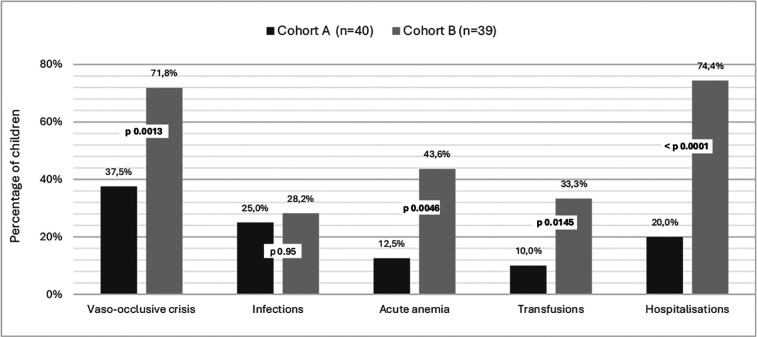
Complications in children with sickle cell disease according to the context of diagnosis. Cohort A are children diagnosed at birth by general screening and followed in the Center for Research and Ambulatory Management of Sickle Cell Disease. Cohort B are children diagnosed after a symptomatic episode consulting to Regional Hospital Center. St-Louis, Senegal.

Regarding complications of the disease, splenomegaly was found in 4 patients (10%) during follow-up. Splenomegaly regression was documented in 3 patients (data missing for 1 child). Clinical findings evocating anemia, mild pallor was documented in 17.5% of patients, moderate pallor in 47.5% and 1 child presenting jaundice. 15 children (37.5%) experienced at least one episode of VOC; 10 in the form of hand-foot syndrome, 3 with other bone locations, and 6 abdominally. 25% (10) of children had infectious complications; 3 abdominal, 2 cutaneous, 2 osseous, and 8 respiratory. 5 (12.5%) children had acute anemia and 4 (10%) were transfused at least once in this context. One child had acute chest syndrome. 8 (20%) children were hospitalized, 3 due to acute anemia, 6 infectious symptoms, 3 VOC, and 1 due to respiratory distress. (Figure 2) No deaths were reported.

**Figure 3:**
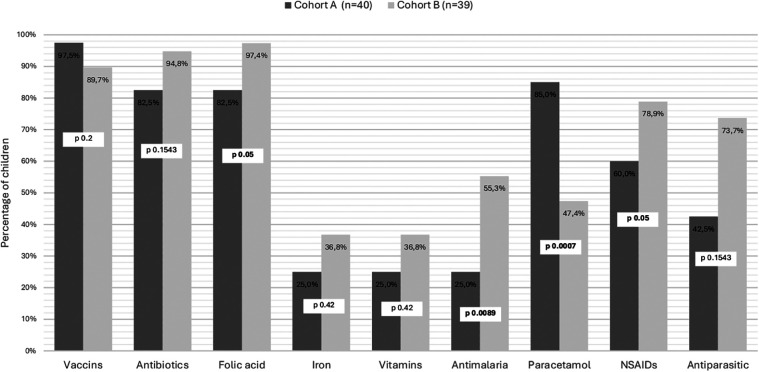
Prescriptions for children with sickle cell disease, according to the context of their diagnosis. Cohort A are children diagnosed at birth by general screening and followed in the Center for Research and Ambulatory Management of Sickle Cell Disease. Cohort B are children diagnosed after a symptomatic episode consulting to Regional Hospital Center. St-Louis, Senegal.

Most children were vaccinated according to the national immunization program (97.5%, 39 children). Figure 3 gives an overview over their respective coverage regarding prophylactic and symptomatic treatment. None of the children had prescribed hydroxyurea.

### 3.2 Cohort B

The cohort B (*n* = 39) consists of children followed at the CHR/SL. Among them, 34 (87.2%) were diagnosed following clinical manifestations, and 5 (12.8%) following a sibling screening; 89.7% were diagnosed directly at CHR/SL, and 10.4% at surrounding centers. Phenotypic distribution was as follows: 94.9% (37) SS, 2.6% (1) SC, 2.6% (1) Sβ. Further epidemiological data of these children are given in Table 1.

Of all parents, 30.8% (12) had prior knowledge of their sickle cell status before their child's diagnosis. Data were collected from 30.8% of fathers and all had an AS profile. All mothers but one could be tested and they all showed an AS profile. Of the 51.3% tested mothers, most of them had a heterozygous profile (90% AS, AC, or other heterozygous form) and 2 mothers (2%) had a homozygous SS profile. In 61.5% of parental couples, consanguinity was present to varying degrees. Parents had an average of 2.76 children, and 36.8% of the children also had at least one sibling with SCD (Table 1).

The clinical manifestations at diagnosis were as follows: acute anemia in 23.5% (8), jaundice in 14.7% (5), hand-foot syndrome in 38.2% (13), infectious symptoms in 50% (17), and VOC in 76.4% (36) [including 38.2% (13) of hand-foot syndrome]. These manifestations could be concomitant. Further comorbidities were malnutrition, three children were known for recurrent infections, one for febrile seizures. One child was born prematurely with perinatal stroke, and two had atopic contexts or asthma.

Regarding complications of the disease, splenomegaly was found in 14 patients (35.9%) during their follow-up. It completely regressed in at least 10 children (4 missing data). About the disease complications, symptoms of anemia were present in 78.9%. The average number of VOC per year was 1.76 for this cohort, with 28 children (71.8%) having at least one VOC episode. Data on VOC localization was incomplete, but at least eight children presented with VOC in the form of hand-foot syndrome. 28.2% (11) of the children had infectious complications: 4 cutaneous, 3 bone, 4 respiratory, and 1 had meningitis. 17 (43.6%) children had acute anemia, and 13 (33.3%) were transfused at least once in this context (see Figure 2). One child had acute chest syndrome. One child exhibited cerebral stroke symptoms. 29 (74.4%) children were hospitalized at least once. Causes of hospitalization are presented in Figure 2. No deaths were observed before the age of 5 years.

Most children in Cohort B were vaccinated according to the national immunization program (89.7%) and rate of preventive measures like antibiotic prophylaxis, mainly with amoxicillin and folic acid were quite high (Figure 3). Prescription for analgesia was predominantly oriented NSAID's in 78.9% and Paracetamol in 47.4%. None of the children had prescribed hydroxyurea.

## 4 Discussion

SCD is a significant public health challenge in Senegal. Despite the technical feasibility and screening programs in neighboring countries, Senegal has yet to implement neonatal screening to identify high-risk patients and integrate them into early clinical follow-up. This study demonstrates the feasibility and potential benefits of neonatal SCD screening by comparing outcomes in a screened cohort with a non-screened cohort up to age 5.

In the two largest maternity wards of St-Louis, general neonatal screening was performed with a coverage of 74%. No published studies assessing the coverage of sickle cell screening in Senegal have been documented in the literature so far. Despite having initiated a pilot newborn screening program, Ghana currently reports a coverage rate of only 3% of annual births. In Nigeria, existing pilot initiatives reach approximately 0.1% of newborns. In the Democratic Republic of Congo, coverage is higher in the city of Kisangani, where it reaches approximately 14.2%; however, nationwide coverage remains much lower, estimated at around 1.4% (25). The high coverage achieved in St-Louis demonstrates the potential impact of structured, routine screening programs and reinforces the importance of moving beyond pilot phases toward nationwide integration to ensure early diagnosis and care for affected newborns.

Regarding methodological considerations, POCTs like HemoTypeSC and Sickle SCAN® are generally reliable but false negatives can occur, particularly in newborns with high levels of fetal hemoglobin or in cases of compound heterozygosity (e.g., HbSC, HbS/β-thalassemia), which may be harder to detect. As said in the introduction section, some POCTs have limited ability to distinguish between certain variants or accurately identify compound forms. False positives, though less frequent, may also occur. Therefore, confirmatory testing using IEF, HPLC, or capillary electrophoresis remains essential. POCTs offer practical advantages in low-resource settings but must be integrated into a broader diagnostic framework with adequate training and quality control.

Children diagnosed at birth had a mean age of 70.48 days at inclusion, close to the average of 60 days in France, where neonatal screening detects cases around day 7, but diagnosis requires confirmation (26). In contrast, children diagnosed during symptomatic episodes had a much higher mean age of 21.9 months. This is comparable to the age at diagnosis reported in European cohorts without neonatal screening (27) and remains earlier than the typical age of diagnosis in Senegal, which is close to 5 years (28). This illustrates that neonatal screening reduces the age of diagnosis and therefore offers a potential window for introducing preventive measures and regular controls.

The cohort identified through neonatal screening showed phenotypic distribution consistent with literature, with a high proportion of SS forms and a low proportion of SC forms (29). The non-screened cohort had a very low proportion of SC forms and was predominantly composed of individuals with homozygous SS disease, consistent with findings from West African populations where the SS phenotype is most prevalent (30). This distribution also reflects the clinical reality that more severe genotypes, such as SS, are more likely to be identified in the absence of systematic screening due to earlier symptomatic presentation (31). As both cohorts were assessed using the same diagnostic methods, the observed differences in phenotypic distribution are unlikely to result from technical variability. Rather, they underscore the limitations of symptom-based diagnosis, which tends to underrepresent milder genotypes such as SC, and highlight the importance of implementing systematic neonatal screening to improve early detection of children with any variants.

Sex distribution varied unexpectedly (given the autosomal recessive transmission) and significantly between the two cohorts, with more female in the children diagnosed at birth. This discrepancy may reflect underlying sex-based biological differences in the progression and severity of SCD. In adulthood, males with SCD are more likely to experience severe complications and higher mortality rates, a pattern often attributed to hormonal influences (potential protective effects of estrogens) (32, 33). Notably, emerging evidence suggests that sex-based disparities in disease severity may already be present in childhood, although the mechanisms remain incompletely understood (32). Even prior to puberty, females tend to exhibit higher estrogen levels and more robust immune responses than males, which may confer a degree of protection and delay the onset or severity of symptoms (34). These early biological advantages could partly account for the greater representation of females in the birth-screened cohort. More broadly, sex-related differences in pediatric mortality vary across regions and socioeconomic contexts. For instance, under-five mortality rates are higher among females in India, whereas in high-income countries, early childhood mortality is typically higher among males (35). This variation underscores the complex interplay of biological, socioeconomic, healthcare, and cultural factors in shaping sex-based differences in disease outcomes and the sex distribution observed within our cohorts.

Regarding clinical complications, more severe symptoms, such as vaso-occlusive crises (VOC), anemia, and the need of transfusions and hospitalizations, were significantly more present in the non-screened cohort. Comparative data on clinical findings, especially in similar settings, remain limited. However, older children, who often manage their crises independently, tend to consult less frequently but present with more severe symptoms (36). A U.S. study reported that 90% of adults with SCD had experienced at least one VOC in the previous year (37). The high VOC-prevalence may be attributed to older age, lower fetal hemoglobin levels, and more severe phenotypes in this cohort. Early diagnosis can significantly reduce disease complications, as it is well documented in the literature. The WHO estimates that up to 70% of SCD-related deaths in Africa could be prevented with appropriate medical care, and adherence to clinical guidelines could reduce mortality by a factor of 10 in children under 5 (38, 39).

Several countries with diverse healthcare systems have implemented newborn screening programs for sickle cell disease, and few studies evaluated its impact and feasibility, offering valuable points of comparison to our findings in Senegal. In Catalonia, the systematic screening program carried out between 2015 and 2022 enabled early detection of cases, with a significant improvement in clinical follow-up and a reduction in acute events during early childhood (17). In Quebec, 10 years of universal newborn screening led to a notable reduction in hospitalization rates and emergency department visits, demonstrating the positive impact of early diagnosis on disease morbidity (40). In the Democratic Republic of Congo, newborn screening initiatives have been launched in cities like Kindu, where pilot studies have shown the feasibility and effectiveness of using rapid diagnostic tests to identify newborns with sickle cell disease (25).

We observed excellent adherence to the national vaccination schedule in both cohorts, with vaccinations being administered at accessible regional health centers. This raises considerations regarding the optimal implementation sites for neonatal screening programs.

Both cohorts showed a high rate of antibiotic prophylaxis coverage, in line with international standards of care (41). The rate was slightly higher in the non-screened cohort. This could reflect both greater tendency to prescribe treatment in clinically ill children and increased adherence by families when symptoms are evident. In asymptomatic children diagnosed at birth, hesitation from both providers and caregivers may reduce early and consistent initiation of prophylaxis. These findings underline the importance of reinforcing guideline-based management and caregiver education following neonatal diagnosis. Additionally, children diagnosed with symptomatic sickle cell anemia were significantly more likely to receive NSAIDS, folic acid supplementation and antimalarial treatments. This trend could reflect a more intensive management approach for children presenting with more severe symptoms. In contrast, children identified early through neonatal screening more commonly relied on paracetamol, a first-line analgesic, for pain management. Some children in these two cohorts were receiving iron and vitamin supplements, despite iron not being recommended as an effective preventive treatment and potentially being considered harmful (41). None of the children had a prescribed treatment of hydroxyurea, which was not part of local clinical practice, despite being commonly used elsewhere and even included in local guidelines in other sub-Saharan African countries (42).

Neonatal screening also enables early genetic counselling for parents. Parental knowledge of SCD was lower in the screened cohort, highlighting the need for better information and parental instruction. Consanguinity rates were high in both cohorts, with over half of patients from consanguineous marriages, contributing to the prevalence of the SS genotype. These results are similar to those reported by Boiro et al. and Abderrahim et al. (48) in Fès, Morocco, who found consanguinity rates of 50% and 57.1%, respectively (10, 43). This underscores the role of genetic counselling in public health efforts to address SCD (44). Additionally, neonatal screening extends beyond children, as other family members were also diagnosed following a positive neonatal screening. Moreover, neonatal screening serves as a preventive tool for a broader population. By identifying the status of parents through the diagnoses of their children, it becomes possible to implement preventive measures not only for the parents but also for future pregnancies.

One of the drawbacks of our study is the urban environment. The broader population in rural settings have potentially lower access to healthcare centers and transportation costs may influence the degree of addressing children during symptomatic episodes.

Due to challenges in data accessibility and the relatively small cohort size, the overall quality of our study results is limited. Nonetheless, the findings offer valuable insights, and a prospective follow-up of these children could significantly enhance the robustness and reliability of the results.

Efforts to reduce infant mortality in West Africa should be focused on early disease screening, including SCD. Government support is crucial for sustaining neonatal screening, which is cost-effective in areas where SCD incidence exceeds 0.2%–0.3% (19, 45). This threshold is surpassed in many sub-Saharan countries, making nationwide systematic neonatal screening programs both relevant and economical. Wealthier families often have better access to care due to higher education and financial resources. A study on barriers to healthcare access among women in Sub-Saharan Africa highlights that poorer households face significantly more obstacles, such as lower educational levels and limited autonomy in health-related decision-making (46).

## 5 Conclusion

This study shows feasibility and benefits of neonatal screening for SCD in Senegal. Indeed, neonatal screening reduced the average age at diagnosis, creating opportunities for timely interventions, and is associated with fewer severe complications compared to children diagnosed during symptomatic episodes. While both cohorts maintained adequate preventive measures, including antimicrobial coverage and high vaccination rates, the symptomatic cohort required more intensive therapeutic interventions. This study also underscores the importance of genetic counselling and public health interventions, such as improving parental awareness of their own sickle cell status and addressing the high rates of consanguinity, which contribute to the transmission of SCD. Neonatal screening holds significant relevance in providing benefits for families and improving health outcomes for future generations. Its implementation was shown to be feasible within existing healthcare infrastructures, though barriers to implementing systematic screening remain, including funding, equipment, and trained personnel. This study shows that neonatal screening in existing health care frameworks in Senegal is feasible and might reduce SCD-related morbidity and mortality, aligning with the WHO's recommendations for high-prevalence regions (47).

## Data Availability

The raw data supporting the conclusions of this article will be made available by the authors, without undue reservation.
